# Traumatic brain injury-associated epigenetic changes and the risk for neurodegenerative diseases

**DOI:** 10.3389/fnins.2023.1259405

**Published:** 2023-09-19

**Authors:** Paul Smolen, Pramod K. Dash, John B. Redell

**Affiliations:** Department of Neurobiology and Anatomy, McGovern Medical School, University of Texas Health Science Center, Houston, TX, United States

**Keywords:** acetylation, Alzheimer’s disease, dementia, feedback loop, epigenetics, methylation, Parkinson’s disease, encephalopathy

## Abstract

Epidemiological studies have shown that traumatic brain injury (TBI) increases the risk for developing neurodegenerative diseases (NDs). However, molecular mechanisms that underlie this risk are largely unidentified. TBI triggers widespread epigenetic modifications. Similarly, NDs such as Alzheimer’s or Parkinson’s are associated with numerous epigenetic changes. Although epigenetic changes can persist after TBI, it is unresolved if these modifications increase the risk of later ND development and/or dementia. We briefly review TBI-related epigenetic changes, and point out putative feedback loops that might contribute to long-term persistence of some modifications. We then focus on evidence suggesting persistent TBI-associated epigenetic changes may contribute to pathological processes (e.g., neuroinflammation) which may facilitate the development of specific NDs – Alzheimer’s disease, Parkinson’s disease, or chronic traumatic encephalopathy. Finally, we discuss possible directions for TBI therapies that may help prevent or delay development of NDs.

## Introduction

Epigenetic information includes reversible modifications to DNA, or to DNA-associated histone proteins involved in regulating gene expression (Fitz-James and Cavalli, 2022). Long-lasting epigenetic changes occur in many disease states (Lardenoije et al., 2018; Cavalli and Heard, 2019; Bertogliat et al., 2020) and play important roles in aging and long-term memory (Kim and Kaang, 2017; Bellver-Sanchis et al., 2021). Epigenetic modifications and their putative impact on the pathology of neurodegenerative diseases (NDs), including Alzheimer’s disease (AD), and Parkinson’s disease (PD), have been extensively studied (Pavlou and Outeiro, 2017; Berson et al., 2018; Stoccoro and Coppedè, 2018; Bennett et al., 2019; Nikolac Perkovic et al., 2021; Lee et al., 2023). Fewer studies, however, have examined the nature and persistence of epigenetic changes arising from traumatic brain injury (TBI). For some NDs and dementias; such as AD, PD, and chronic traumatic encephalopathy (CTE); evidence suggests TBI (including repeated mild TBI) is a risk factor (Goldman et al., 2006; Gardner et al., 2015; Gardner and Yaffe, 2015; Delic et al., 2020; Schneider et al., 2021; Brett et al., 2022; Graham et al., 2022; Mielke et al., 2022). Epigenetic modifications observed after TBI have also been observed in these NDs. Some changes correlate with persistent neuroinflammation, which commonly follows TBI and may predispose for NDs up to years later (Faden and Loane, 2015; Dams-O'Connor et al., 2016; Brett et al., 2022). These modifications can potentially be pharmacologically targeted to prevent or delay NDs, by targeting the enzymes that add (epigenetic writers), remove (erasers), or decode (readers), epigenetic modifications (Kelly et al., 2010; Cheng et al., 2019; Majchrzak-Celińska et al., 2021).

For brevity, we focus on two common modification types, DNA methylation and histone acetylation. These modifications associate with NDs and TBI and could serve as therapeutic targets (Bruno et al., 2022).

### DNA methylation and histone acetylation

DNA methyltransferases (DNMTs) are writer enzymes that add a methyl (-CH_3_) group to a cytosine base (Okano et al., 1999; Jin and Robertson, 2013; Coppede, 2022), commonly at 5′-cytosine-guanine-3′ (CpG) dinucleotides in a promoter region. Cytosine methylation primarily represses transcription (Cedar and Bergman, 2009), although there are activation examples (Harris et al., 2018; Rauluseviciute et al., 2020). Methylated DNA recruits readers (Fitz-James and Cavalli, 2022) that facilitate local chromatin compaction and can recruit other writers (Bennett and Licht, 2018; Schmidt et al., 2020). Methylation is reversed by erasers of the ten-eleven translocation (Tet) family (Yamaguchi et al., 2013). Most intracellular methylation reactions utilize the universal methyl donor S-adenosyl methionine (SAM; Landgraf et al., 2016), which can be derived from dietary methionine and from folate (vitamin B9). Vitamins B2 and B12, and choline, also help maintain SAM levels and methylation homeostasis (Abbasi et al., 2017; Froese et al., 2019; McNulty et al., 2019).

Each nucleosome of eukaryotic chromatin consists of a histone octamer wound by ~2 turns of DNA (Kornberg, 1974). Attraction between DNA and positively charged histones tends to compactify chromatin, hindering transcription complex access to the promoter (Bartke and Kouzarides, 2011). Acetylation of histone lysine (Gräff and Tsai, 2013) or arginine (Cura and Cavarelli, 2021) residues by acetyltransferase writers neutralizes positive charge, facilitating gene expression. Specificity is mediated by readers that identify patterns of acetylated residues and modulate transcription (Xu et al., 2017; Poulard et al., 2021; Chen et al., 2022). Other histone modifications include methylation, which can be long-lasting (Fitz-James and Cavalli, 2022); phosphorylation, and ubiquitination. Roles of these modifications following TBI have not been well characterized, thus we mainly discuss acetylation.

### TBI as a risk factor for neurodegenerative disease

We focus on three NDs for which there is evidence that TBI is a risk factor, AD, PD, and CTE. For AD, a meta-analysis of 15 case–control studies (Fleminger et al., 2003) and a veteran cohort study (Plassman et al., 2000) identified TBI as a risk factor. For PD, TBI is repeatedly reported as a risk factor (Bower et al., 2003; Lee et al., 2012; Jafari et al., 2013; Delic et al., 2020; although see Rugbjerg et al., 2008; Marras et al., 2014). CTE is a tauopathy with progressive accumulation of Tau protein (McKee et al., 2009; Stein et al., 2014) and has been delineated as a consequence of repeated mild TBI (Gardner and Yaffe, 2015; Smith et al., 2019; McKee et al., 2023). A relationship was found in American football players (McKee, 2020), with CTE-related pathology reported in most players’ post-mortem brains (Mez et al., 2017). Other contact sports, and military injuries, are a risk factor for CTE and likely other ND-related dementias (Chauhan, 2014; Johnson et al., 2017; Smith et al., 2019; Delic et al., 2020).

## Epigenetic modifications contribute to TBI pathology

TBI commonly engenders life-long consequences (Masel and DeWitt, 2010) including cognitive impairments. Experimental TBI, and human studies, have begun exploring post-TBI epigenetic changes (Wong and Langley, 2016; Mateen et al., 2017; Nagalakshmi et al., 2018; Bertogliat et al., 2020). In a rat blast injury model, hippocampal DNA methylation was increased 2 weeks post-injury, as was expression of the writers DNMT1 and DNMT3b (Bailey et al., 2015). Other murine TBI models exhibited hypomethylated as well as hypermethylated loci in cortex (Haghighi et al., 2015; Meng et al., 2017). *Serotonin N-acetyltransferase (Aanat)* was hypermethylated and downregulated (Haghighi et al., 2015), reducing conversion of serotonin to melatonin, possibly contributing to insomnia (Cruz-Sanabria et al., 2023). The *superoxide dismutase 2 (Sod2)* promoter was hypermethylated and its expression downregulated, which could increase oxidative stress (Balasubramanian et al., 2021a). In amygdala, enhanced DNMT activity correlated with *brain-derived neurotrophic factor* (*Bdnf*) methylation and reduced expression (Sagarkar et al., 2017). After repeated TBI, *mitofusin 2 (Mfn2)* hypermethylation and repression triggers mitochondrial dysfunction (Kulkarni et al., 2023). Hypomethylation in perilesional microglia was associated with inflammation (Zhang et al., 2007). DNA methylation changes, most commonly hypomethylation, were found in blood cells following human TBI (Bahado-Singh et al., 2020).

Histone hypoacetylation has been observed in murine cortex post-TBI (Gao et al., 2006) and in hippocampus (Kumari et al., 2023). Increased hippocampal histone deacetylase (HDAC) activity has also been observed (Sagarkar et al., 2019) as was *neuropeptide Y* promoter hypoacetylation (Balasubramanian et al., 2021b). TBI was reported to upregulate HDAC2-5 and HDAC11 (Sagarkar et al., 2019) and downregulate HDAC4-5 (Kamal et al., 2022). HDAC2 upregulation is of interest because its activity has been implicated in AD (see below). It is not yet known if altered histone acetylation persists chronically post-TBI.

Overall, substantial epigenetic changes are induced by TBI. However, only a few changes correlate with data suggesting long-term persistence of these changes or links to development of NDs years later. We discuss links that, although hypothetical, are plausible from data. Thus, below, we do not comprehensively discuss epigenetic changes associated with NDs, but focus on a subset of changes that could be linked to a preceding TBI.

## Hypothetical epigenetic links with specific NDs

### Epigenetic changes are associated with persistent neuroinflammation

Inflammatory biomarkers correlate with cognitive impairment in AD patients (de Oliveira et al., 2021), and persistent neuroinflammation is one hypothesized driver of AD development (see below). Genetic polymorphisms associated with immune system regulation, such as in *TREM2*, *CR1*, and *APOE*, enhance AD risk and correlate with increased neuroinflammation (Karch and Goate, 2015; Lee et al., 2018; de Oliveira et al., 2021). Neuroinflammation and reduced microglia phagocytic function increase Aβ deposition and Tau hyperphosphorylation (Kitazawa et al., 2005; Lee et al., 2008; Li et al., 2022). In turn, Tau hyperphosphorylation promotes Tau aggregation (Limorenko and Lashuel, 2021), plausibly contributing to CTE. In mouse tauopathy, microglia activation preceded deposition of Tau neurofibrillary tangles (Yoshiyama et al., 2007). The cytokine interleukin-1β enhances Tau phosphorylation (Ghosh et al., 2013; Collins-Praino and Corrigan, 2017). Indeed, some data may support a hypothetical positive feedback loop with reciprocal activation of inflammation and Tau hyperphosphorylation/aggregation. Secretion of hyperphosphorylated Tau enhances glia overactivation and cytokine production, and neuroinflammation, in turn, enhances Tau phosphorylation (Lee et al., 2010; Laurent et al., 2018; Fesharaki-Zadeh, 2019; Al-Ghraiybah et al., 2022). Chronic neuroinflammation may also predispose to PD (Rasheed et al., 2021). Inflammation upregulates caspase-1 which in turn increases α-synuclein aggregation (Wang et al., 2016).

Following TBI, neuroinflammation, characterized in part by microglia overactivation, can persist for many years (Ramlackhansingh et al., 2011; Johnson et al., 2013; Coughlin et al., 2015; Makinde et al., 2017; Risbrough et al., 2022) and DNA hypomethylation has been identified in microglia that can enhance neuroinflammation (Zhang et al., 2007). However, does persistent neuroinflammation post-TBI result, at least in part, from persistence of epigenetic changes? A recent study may suggest a link. In mouse cortex, up to 2 years post-TBI, genes in the complement activation and effector pathways were upregulated (Toutonji et al., 2021). Complement inhibition ameliorated this upregulation. These data suggest a hypothetical positive feedback loop between complement activity, persistent inflammation, and transcription. DNA hypomethylation after TBI in microglia suggests such feedback may involve epigenetic modifications. Hippocampal DNA hypomethylation is also reported in AD patients (Chouliaras et al., 2013).

### Additional epigenetic changes may predispose to Alzheimer’s disease

Currently ~6 million Americans live with AD, expected to increase to ~13 million by 2050 (Alzheimer’s Association, www.alz.org). AD correlates with neuronal accumulation of toxic amyloid β (Aβ), with some evidence for causation (Selkoe and Hardy, 2016; Abu Hamdeh et al., 2018; Hampel et al., 2021). Alternative hypotheses for AD causation include accumulation of toxic Tau protein as a primary driver (Guo et al., 2017; Nasb et al., 2023), cumulative oxidative stress (Roy et al., 2023), or persistent inflammation and glial senescence (Lau et al., 2023).

Following human TBI, diffuse Aβ plaques have been observed to accumulate in brain, up to decades post-TBI (Roberts et al., 1991; Johnson et al., 2012; Scott et al., 2016). Aβ accumulation is observed in only a minority of post-TBI human brains, and may correlate with genetic susceptibility (Roberts et al., 1994; Nicoll et al., 1995; DeKosky et al., 2007; Johnson et al., 2009; Smith and Stewart, 2018). Following human TBI, methylation changes in or near the amyloid precursor protein (*APP*), *MAPT* (encoding Tau protein isoforms), and neurofilament genes (*NEFH, NEFM, and NEFL*) have been reported in brain (Abu Hamdeh et al., 2021). Thus, epigenetic upregulation of APP expression, or *MAPT*, may play a role in any post-TBI Aβ or Tau accumulation. However, these changes have not yet been shown to be long-lasting or to upregulate expression. Thus, more research is necessary to investigate links between epigenetic changes post-TBI and subsequent AD, and investigate whether pathology is driven primarily by Aβ or Tau accumulation, or oxidative stress, and/or inflammation.

Hypomethylation of *presenilin 1* (*PSEN1*) correlates with AD, and with increased PSEN1 expression, which may indirectly enhance production of toxic Aβ (Monti et al., 2020). It would be of interest to examine *PSEN1* epigenetic modifications at late time points post-TBI. In contrast, repeated TBI hypermethylates and decreases expression of *Sod2* in murine hippocampus (Balasubramanian et al., 2021a). This decrease could lead to oxidative damage, increasing risk for NDs including AD. DNMT inhibition normalized *Sod2* methylation and expression and ameliorated neurodegeneration and learning deficits.

### Parkinson’s disease

Neuronal and glial epigenetic changes characterize PD (Lee et al., 2023). Hypomethylation in/near the *α-synuclein* gene (*SNCA*) occurs in PD patients and enhances *SNCA* expression (Matsumoto et al., 2010). Consequent α-synuclein accumulation may accelerate PD (Kontopoulos et al., 2006). In turn, α-synuclein was observed to interact with, and mislocalize, DNMT1 (Desplats et al., 2011) which may contribute to hypomethylation and further activation of *SNCA*. These interactions constitute a putative positive feedback loop that could contribute to late α-synuclein accumulation, and consequent PD, following an initial increase due to TBI. α-synuclein has occasionally been observed to increase post-TBI. In rat models, α-synuclein was increased in rat *substantia nigra* (SN) 60 days post-TBI (Acosta et al., 2015), and α-synuclein aggregation was enhanced in SN and striatum (Acosta et al., 2019). In two studies, the majority of postmortem human brains with a history of recent severe TBI showed α-synuclein accumulation (Ikonomovic et al., 2004; Uryu et al., 2007). However, another study did not find α-synuclein accumulation in brain samples from 53 individuals with a history of remote TBI (Postupna et al., 2021).

### Chronic traumatic encephalopathy

Human TBI can induce accumulation of Tau (Collins-Praino and Corrigan, 2017; Clark et al., 2021). In the Sydney Brain Bank, observed prevalence of CTE pathology was relatively low (0.79%), suggesting single TBI may be unlikely to cause long-lasting Tau accumulation or CTE, with repeated TBI plausibly required (McCann et al., 2022). In rats, TBI can induce a phosphorylated Tau species that correlates with neuropathology (Hintermayer et al., 2020). Following a TBI-induced Tau increase, positive feedback favoring further Tau phosphorylation could lead to CTE tauopathy. A recent study (Wang et al., 2021) suggests epigenetics could drive such a positive feedback loop. In P301S Tau mutant mouse cortex, Tau is hyperphosphorylated and aggregates, and activity of euchromatic histone-lysine N-methyltransferase (EHMT), which methylates histones, is elevated. EHMT inhibition reduced hyperphosphorylated Tau, supporting the existence of positive feedback in which EHMT activity elevation indirectly drives further Tau phosphorylation. Alternatively, EHMT could mediate such feedback via a non-histone substrate.

Table 1 lists representative genes found to be epigenetically modified either after TBI, or in an ND (either in humans or in rodent TBI or ND models), putative links to other pathologies, and whether the modification is persistent, if known.

**Table 1:** Representative genes epigenetically altered due to TBI or in AD or PD.

Gene(s) and reference	Modified after TBI, or in an ND?	Reference(s) suggesting gene expression changes, or mutations, may be linked to another pathology (ND or TBI)	Is change assessed short-term (here ≤1 month) or long-term?
*APP* (Abu Hamdeh et al., 2021)	TBI	AD (Andrade-Guerrero et al., 2023).	Short-term (<14 days)
*MAPT* (Abu Hamdeh et al., 2021)	TBI	Frontotemporal dementia and AD (Coppola et al., 2012; Strang et al., 2019)	Short-term (<14 days)
*Bdnf* (Sagarkar et al., 2017; Treble-Barna et al., 2021)	TBI	AD (Song et al., 2015), PD (Scalzo et al., 2010)	Short-term (≤30 days)
*Tfam* (Balasubramanian et al., 2021c)	TBI	AD, PD (Kang et al., 2018)	Short-term (30 days)
*Sod2* (Balasubramanian et al., 2021a)	TBI	AD (Castora et al., 2022)	Short-term (30 days)
*Igf1* (Corne et al., 2021)	TBI	AD (Dato et al., 2023)	Short-term (≤3 weeks)
*Mfn2* (Kulkarni et al., 2023)	TBI	AD (Castora et al., 2022)	Short-term (30 days)
*Neuropeptide Y* (Balasubramanian et al., 2021b)	TBI	PD (Cannizzaro et al., 2003)	Short-term (30 days)
*RGMA* (Liu et al., 2022)	TBI	PD (Korecka et al., 2017)	Short-term (≤5 days)
*NEFL, NEFM* (Abu Hamdeh et al., 2021)	TBI	AD (Kittur et al., 1994)	Short-term (<14 days)
*NEFH* (Abu Hamdeh et al., 2021)	TBI		Short-term (<14 days)
*Aanat* (Haghighi et al., 2015)	TBI		Long-term (8 months)
*Per3* (Haghighi et al., 2015)	TBI	3 x TG AD (Bellanti et al., 2017)	Long-term (8 months)
*Park7* (Haghighi et al., 2015)	TBI	PD (Huang and Chen, 2021)	Long-term (8 months)
*PSEN1* (Monti et al., 2020)	AD	PD, frontotemporal dementia (Yang et al., 2023), TBI (Thangavelu et al., 2020)	Long-term (post-mortem)
*SNCA* (Jowaed et al., 2010; Matsumoto et al., 2010)	PD	TBI (Ikonomovic et al., 2004; Uryu et al., 2007; Acosta et al., 2015, 2019)	Long-term (post-mortem)

List of genes, and noted links, is not meant to be complete. Each cited study in the first column examined either TBI or a single ND, thus each row has a single entry in the second column. In the last column, the time frame for change assessment refers to the primary reference in the first column.

## Therapeutic directions that may inhibit ND development

Considering the above putative epigenetic links between TBI and NDs suggests strategies that might prevent or delay NDs.

### Methyl donors

Blood levels of SAM and methionine are decreased in TBI patients (Dash et al., 2016). S-adenosyl-L-methionine decarboxylase, which synthesizes SAM, is decreased in rat motor cortex post-TBI, which may contribute to reduced epigenetic methylation (Henley et al., 1997). SAM administration alters DNA methylation in macrophages and reduces inflammation (Pfalzer et al., 2014). These data suggest SAM might ameliorate some adverse TBI sequelae (Schieffler and Matta, 2022; Zima et al., 2022). In one clinical trial, administration of SAM post-concussion correlated with a 77% reduction in adverse clinical scores, compared to a 49% reduction with placebo (Bacci Ballerini et al., 1983). Global DNA hypomethylation appears to correlate with long-lasting neuroinflammation (Gonzalez-Jaramillo et al., 2019) which might be alleviated by SAM. In a murine AD model, SAM reduced Aβ deposition and rescued cognitive deficits (Do Carmo et al., 2016).

Thus, treatment or dietary supplementation by SAM or B vitamins might ameliorate short-term TBI sequelae and provide some ND protection. However, there are caveats. Hypermethylation of *SOD2* occurs post-TBI and suppresses its expression as noted above. Methyl donors might further suppress *SOD2* expression. Supplementing mouse fibroblast cultures with SAM was reported to overproduce adenine and methylthioadenosine, leading to toxicity (Fukumoto et al., 2022). It will be important to examine whether different doses of SAM or alternative methyl donors, such as B vitamins, show fewer potential adverse effects. Epigenetics may not underlie all therapeutic effects of methyl donors. SAM is a cofactor for enzymes that do not catalyze methylation but associate with human diseases (Landgraf et al., 2016). The potential therapeutic usefulness of methyl donors is further discussed in Coppede (2022). It is important to note that for methyl donors as well as other potential therapies discussed here, our discussion is relatively speculative, insofar as no clinical results have been published demonstrating the efficacy, for any ND, of these potential treatments. As of August 2023, clinical trials are examining the efficacy of B vitamin supplementation, exercise, and complement inhibition as therapies post-TBI (ClinicalTrials.gov).

In case of adverse effects, more targeted modifications might be preferred. Specific isoforms of demethylating (or methylating) enzymes involved might be targeted. Or, methylation might be altered at specific gene loci. This could be useful if, for example, a treatment could break positive feedback in which aberrant methylation of a locus, induced by TBI, becomes self-sustaining. This direction would require development of vectors crossing the blood–brain barrier, plausibly using CRISPR/Cas-based gene editors (Urbano et al., 2019; Zou et al., 2022).

### Inhibition of chronic neuroinflammation

As discussed above, positive feedback may occur between neuroinflammation and Tau aggregation/propagation (Laurent et al., 2018; Fesharaki-Zadeh, 2019; Al-Ghraiybah et al., 2022). Post-TBI anti-inflammatory treatment might break such a feedback loop (Selvaraj et al., 2021; Kip and Parr-Brownlie, 2022; McGovern et al., 2022; Smith et al., 2022). Gene expression in the complement activation and effector pathways was upregulated up to 2 years post-TBI in mouse (Toutonji et al., 2021). Thus, hypothetically, complement inhibitor therapy may ameliorate neuroinflammation and ND development.

### Histone deacetylase inhibition

Although late persistence of post-TBI histone hypoacetylation has not been established, it would be interesting to explore possible links with ND development. Following rodent TBI, spatial and fear learning and other cognitive measures are improved by HDAC inhibitors (HDACIs; Dash et al., 2009; Shein et al., 2009; Yu et al., 2013; Tai et al., 2014; Sagarkar et al., 2019) and microglia inflammatory responses reduced (Zhang et al., 2008). The HDACI Scriptaid improves neuronal survival and other aspects of pathology (Wang et al., 2013; Meng et al., 2020). HDAC inhibition may also decrease expression of pro-inflammatory factors in neurons and immune cells (Dai et al., 2021; Ghiboub et al., 2021). These data suggest HDAC inhibition may inhibit the process – neuroinflammation – that currently appears to provide the strongest long-term link between TBI and subsequent NDs. HDAC2 activity is increased in AD patient brains, and HDAC2 knockdown in a mouse neurodegeneration model rescued learning and memory impairments and related gene expression (Gräff et al., 2012). Thus, HDACIs specific to HDAC2 may be of particular interest.

Vorinostat, a relatively broad-spectrum HDAC inhibitor, is in a clinical trial as a potential AD treatment (Bondarev et al., 2021). However, there is concern regarding potential toxicity of long-term HDAC inhibition in humans, with more study of targeted inhibitors needed (Coppede, 2022). One study suggests HDAC1 protects against neurodegeneration (Patnaik et al., 2021) so that an HDAC1 activator might also be therapeutic.

### Histone methylation inhibition

As discussed above positive feedback may sustain Tau hyperphosphorylation and histone methylation, and inhibiting histone n-lysine methyl transferase (EHMT) reduced hyperphosphorylated Tau (Wang et al., 2021). It would be of interest to investigate whether post-TBI Tau aggregation could be reduced in this way. This might help prevent CTE, or other tauopathies.

### Exercise and diet

Evidence suggests habitual exercise can prevent or delay NDs and reduce chronic neuroinflammation (Coppede, 2022; Ribarič, 2022; Sujkowski et al., 2022). Numerous epigenetic changes in rodent brain tissue have been observed following endurance exercise (Fernandes et al., 2017). Prominent are altered histone acetylation, enhanced expression of DNA demethylases, and DNA methylation changes at/near genes including *Bdnf* (Sellami et al., 2021; Xu et al., 2021). Levels of BDNF and another growth factor, IGF-1, are enhanced by exercise (Vaynman et al., 2004; Ding et al., 2006), which would help to counter an age-associated BDNF decrease (Xu et al., 2021). Enhanced growth factor activity likely helps maintain neural circuits, improving TBI recovery and reducing ND susceptibility. Thus an exercise program for TBI survivors might delay or prevent NDs.

Finally, studies have found a “Mediterranean-style” diet, rich in fruits and vegetables, and in flavonoids and antioxidants, to associate with a reduced risk of AD and other NDs (Holland et al., 2020; Migliore and Coppede, 2022), suggesting recommendation of these diets post-TBI.

## Conclusion

Studies of epigenetic changes post-TBI, and their possible relationships with subsequent ND development, are beginning to provide suggestions for therapeutic directions that may prevent or reduce the risk for human NDs. But many gaps in knowledge need to be addressed. Prominently: (1) For most cases, the possible persistence over months or years of specific epigenetic changes that correlate with TBI has not been examined, thus their potential relevance to subsequent ND development cannot be assessed. (2) If changes are found to be persistent, what are the mechanisms underlying persistence, e.g., positive feedback? Mechanistic understanding may prove essential to develop therapies. (3) There is a profound lack of clinical studies examining the long-term effects of potential therapies.

Overall, we are at very early stages in understanding the epigenetic effects of TBI and their relationship with NDs. This active field is primed to deliver numerous insights into epigenetic events, and treatments, in years to come.

## Data availability statement

The original contributions presented in the study are included in the article/supplementary material, further inquiries can be directed to the corresponding author.

## Author contributions

PS: Conceptualization, Investigation, Writing – original draft, Writing – review and editing. PD: Conceptualization, Supervision, Writing – review and editing, Funding acquisition. JR: Conceptualization, Project administration, Supervision, Writing – review and editing.

## Funding

The author(s) declare financial support was received for the research, authorship, and/or publication of this article. This study was supported in part by an NIH grant (NS121261; PD) and by the Nina and Michael Zilkha Distinguished Chair in Neurodegenerative Research (PD).

## Conflict of interest

The authors declare that the research was conducted in the absence of any commercial or financial relationships that could be construed as a potential conflict of interest.

## Publisher’s note

All claims expressed in this article are solely those of the authors and do not necessarily represent those of their affiliated organizations, or those of the publisher, the editors and the reviewers. Any product that may be evaluated in this article, or claim that may be made by its manufacturer, is not guaranteed or endorsed by the publisher.

## Abbreviations

Aβ: Amyloid β
AD: Alzheimer’s disease
APP: Amyloid precursor protein
CTE: Chronic traumatic encephalopathy
DNMT: DNA methyl transferase
EHMT: Euchromatic histone-lysine N-methyltransferase
HDAC: Histone deacetylase
HDACI: HDAC inhibitor
LTP: Long-term potentiation
ND: Neurodegenerative disease
PD: Parkinson’s disease
PSEN1: Presenilin 1
SNCA: α-Synuclein
SAM: S-Adenosyl methionine
Sod2: Superoxide dismutase 2
TBI: Traumatic brain injury
